# Permissible Cathodic Polarization Levels for Underground Stainless Steel Structures in Cathodic Protection Systems

**DOI:** 10.3390/ma19132813

**Published:** 2026-07-02

**Authors:** Mateusz Gniady, Krzysztof Żakowski, Stefan Krakowiak, Michał Szociński, Krzysztof Wzorek, Chengtao Wang

**Affiliations:** 1 Department of Corrosion and Electrochemistry, Faculty of Chemistry, Gdansk University of Technology, 11/12 Gabriela Narutowicza Street, 80-233 Gdansk, Poland; s185174@student.pg.edu.pl (M.G.); krzysztof.zakowski@pg.edu.pl (K.Ż.); stefan.krakowiak@pg.edu.pl (S.K.); 2 Implementation Doctoral School, Gdansk University of Technology, 11/12 Gabriela Narutowicza Street, 80-233 Gdansk, Poland; krzysztof.wzorek@pg.edu.pl; 3 School of Electrical and Control Engineering, Xuzhou University of Technology, Xuzhou 221018, China; chengtaowang@xzit.edu.cn

**Keywords:** cathodic protection, stainless steel, critical potential of hydrogen evolution

## Abstract

Excessive cathodic polarization of underground stainless steel structures results in hydrogen evolution, increasing the risk of hydrogen embrittlement and the disbonding of protective coatings from the structure’s surface. This study was conducted to determine the critical potential and critical cathodic protection current density at which hydrogen evolution occurs on the surfaces of stainless steel grades 1.4301, 1.4401, 1.4125, and 1.4512, and, for comparison, on carbon steel S235. The tests were carried out in an aqueous solution of synthetic (artificial) soil and in a soil filtrate prepared from a soil sample taken in the vicinity of an existing underground gas pipeline connection with stainless steel fittings. The tests showed that the higher the chromium content in the stainless steel was, the lower (more negative) the hydrogen evolution potential was. In an artificial soil environment, the values of this potential ranged from −1105 mV to −1175 mV vs. copper sulphate electrode (CSE) for steels 1.4301, 1.4401, and 1.4125, which contain more than 16% of chromium. For steel 1.4512, containing 12% of chromium, the hydrogen evolution potential was −1050 mV. For comparison, for S235 carbon steel, the hydrogen evolution potential was −1135 mV. The critical cathodic protection current density ranged from 0.30 A/m^2^ to 0.38 A/m^2^ for all tested stainless steels, whilst for S235 steel, this value was higher, equal to 0.65 A/m^2^. The results obtained indicate that applying the commonly accepted potential criterion for cathodic protection for carbon steels (i.e., polarization to a potential in the range from −0.85 V to −1.1 V vs. CSE) poses no risk of causing excessive cathodic polarization of stainless steel. This is important for the design and operation of cathodic protection systems for complex structures containing galvanically connected carbon steel and stainless steel components.

## 1. Introduction

Stainless steels are one of the most common groups of metal alloys, characterized by high corrosion resistance thanks to the passive layer that forms on their surface. The formation of this layer is possible thanks to a minimum of 10.5% chromium content in the alloy [1]. Corrosion resistance and physical properties such as stress resistance and high temperature resistance make these steels widely used in industry in areas particularly exposed to high corrosion aggressiveness.

In underground structures, stainless steel is used for the construction of gas pipelines, pipelines, and grounding systems [2]. Table 1 presents the selection of steel grades according to EN 10027-2 [3] depending on the soil environment and the location of the structure. Regardless of the type of stainless steel selected, choosing the right grade does not guarantee the complete elimination of all corrosion risks. These risks include those posed by the presence of microorganisms, sulfides, and an acidic environment, as well as the need for protection against stray current corrosion [4,5].

Underground structures made of stainless steel are a significant engineering solution that combines high durability with low maintenance requirements in soil environments. Unlike traditional materials commonly used underground (such as carbon steel with protective coatings or reinforced concrete), stainless steel offers superior corrosion resistance, has good mechanical properties, and allows for reduced cross-sections whilst maintaining strength, which translates into a longer service life for the structure and lower operating costs.

Stainless steels exhibit natural corrosion resistance due to the presence of a passive layer and can ensure a very long service life for underground structures. However, this resistance can deteriorate significantly in underground conditions [6]. Variations in soil environment parameters—such as chloride content, sulphates, moisture content, pH, resistivity and microbial activity—can lead to the destabilization of the passive layer and the initiation and development of localized forms of corrosion [7,8], including pitting, crevice corrosion, stress cracking corrosion and microbiologically influenced corrosion. These phenomena challenge the traditional assumption that stainless steel is ‘maintenance-free’ and highlight the need for a more precise selection of steel grades, analysis of the soil environment, and the application of appropriate corrosion protection methods. Therefore, cathodic protection is also used for the corrosion protection of stainless steel structures, particularly in the case of facilities of strategic importance and with high operational criticality. However, this corrosion protection technology should be treated as part of a hybrid system rather than a stand-alone solution. The benefits of combining protective coatings with cathodic protection are significant, with the coating designed to reduce current demand, whilst cathodic protection compensates for localized damage to the coating and areas with increased environmental aggressiveness [9]. In many underground applications, selecting a stainless steel grade with a higher PREN (Pitting Resistance Equivalent Number) can reduce the need for cathodic protection [10]. However, in aggressive environments, hybrid solutions should be used: stainless steel + coating + cathodic protection.

In the case of structures made of stainless steel, the use of cathodic protection is a subject of much debate and requires a precise design approach. Appropriately selected cathodic protection parameters can effectively reduce the risk of stainless steel degradation in aggressive environments, particularly in soils with a high chloride content or in areas with limited oxygen exchange. From the perspective of stainless steels, cathodic protection must interfere with the material’s passive state. Moderate cathodic polarization can stabilize the passive layer and limit the initiation of pitting in chloride-rich environments, whereas excessive polarization promotes intense hydrogen evolution and may lead to depassivation and accelerated crevice corrosion [11]. Under conditions of cyclic wetting and drying of the soil, changes in moisture content and oxygen availability significantly alter ion transport mechanisms and the course of cathodic reactions, making it difficult to apply simple cathodic protection criteria based solely on the protection potential [12].

Large cathodic polarization results in the thermodynamic stability of hydroxonium ions (H_3_O^+^) and water being exceeded, leading to the evolution of atomic hydrogen at the surface of the cathodically polarized metal [13,14]. In electrolytic environments (soils and natural waters), an excessive reduction in the potential of a cathodically protected structure leads to the dominance of hydrogen evolution reactions, and some of the atomic hydrogen produced is absorbed into the steel, which may initiate hydrogen embrittlement [15,16]. The hydrogen evolution potential depends on the electrochemical reaction environment and on the polarized metal. The hydrogen produced can diffuse into the steel’s crystal structure. Exceeding the ‘safe’ range of cathodic protection potentials and excessive polarization can therefore lead to intense hydrogen evolution, an increased risk of hydrogen embrittlement, and steel cracking [17,18]. Austenitic stainless steels are particularly susceptible to this type of corrosion attack [19,20]. An additional effect of excessive polarization leading to hydrogen evolution may be the delamination of protective coatings designed to safeguard the structure against corrosion [21,22].

The criteria for the cathodic protection of underground structures made of unalloyed and low-alloy steel have been known and applied for many years: the structures are polarized to a potential ranging from −850 mV (the so-called protection potential E_P_) to −1100 mV (the so-called critical potential E_L_) relative to a copper sulphate electrode Cu/CuSO_4_ (CSE)—see standard EN 12954 [23]. This potential criterion is recommended by standards [24,25] and is widely accepted in the practice of corrosion protection for underground structures [26]. However, for the reasons discussed above, this criterion raises concerns regarding stainless steels, particularly in the case of cathodic protection of complex structures [27] where, for design reasons, carbon steel components are galvanically connected to alloy steel components. In such cases, the polarizing current from the anodes also flows to the stainless steels, polarizing their surfaces—and there is a concern that using a polarization level suitable for carbon steel may result in excessive polarization of the stainless steel [28].

Review articles on the behavior of steel under cathodic protection indicate that the classical criteria based on a potential of −850 mV vs. CSE for carbon steels cannot be directly applied to stainless steels, particularly with regard to the critical potential. An analysis of the hydrogen embrittlement properties of stainless steels in a high-pressure hydrogen environment shows that, as the hydrogen concentration in austenitic stainless steels increases, there is a significant reduction in ductility and fatigue life [29]—which is analogous to the situation where hydrogen penetrates the steel as a result of excessive cathodic polarization in the electrolyte.

Historically, research of the cathodic protection of underground structures has focused mainly on carbon steels and concrete reinforcement, whilst the field of its application to stainless steels in the ground is developing at a significantly slower pace. There are significant gaps in the literature: a limited database of long-term operational observations of underground stainless steel components in various soil types, insufficient standardization of guidelines regarding structural design and the selection of alloys for specific environmental scenarios, and the need for advanced numerical models combining corrosion processes with soil properties and mechanics. Currently, the recommendations regarding the use of cathodic protection contained in technical standards and codes specify the requirements and protection potential criteria for non-alloy steel structures in detail, but not for stainless steels. For example, the EN 12954 standard provides only the E_P_ potential value for stainless steels of −450 mV vs. CSE, with the proviso that in each case the protection potential and the critical potential E_L_ should be determined individually based on tests taking into account the steel grade and the environmental conditions of the structure’s operation.

The lack of information in scientific articles and standards regarding critical potentials at which hydrogen evolution occurs on the cathodically polarized surfaces of various grades of stainless steel poses a problem for users of underground stainless steel structures in terms of selecting the optimal operating parameters for cathodic protection systems to avoid the potential risks discussed above.

This publication may help to fill this gap. This study aimed to conduct research to determine the permissible level of cathodic polarization for selected grades of stainless steel and the critical cathodic polarization current density in soil. The results presented can be used as practical recommendations for designers of cathodic protection systems, as well as for personnel operating protection installations, regarding the permissible value of the critical potential, particularly in complex systems containing carbon steel and stainless steel components that are metallically connected to one another.

## 2. Materials and Methods

The hydrogen evolution potential at the metal surface in an aqueous environment is determined mainly by electrochemical methods, which allow the cathodic polarization to be observed and the overpotential of the hydrogen evolution reaction to be determined. Potentiodynamic polarization curves are the most commonly used tool, and such an approach has been employed in this paper. This method involves recording the current–potential characteristics upon a slow, continuous change in the electrode potential within a specified range (electrode potential scanning). Polarization curves allow the determination of the potential at which H_2_ evolution begins and enable analysis of the reaction mechanism and kinetics. Similar analyses can be carried out using the galvanostatic method, in which a direct current of a given magnitude is applied, and the electrode potential is then measured. The current–potential potentiostatic curves, obtained for a specific range of forced polarization current values, can be analyzed. This approach has not been utilized in this paper.

Additionally, we conducted an exposure of samples under cathodic polarization conditions to determine the corrosion rate of the samples polarized to selected values of potential. In this experiment, the corrosion rate was calculated based on the gravimetric method.

### 2.1. Solutions

Two solutions were prepared for the tests to reflect the conditions of cathodic polarization in soil. The first one, artificial soil solution, was prepared on the basis of [30]. Table 2 lists the chemical compounds used to obtain 5 dm^3^ of solution. The reagents were weighed on a RADWAG^®^ AS 310.R2 analytical balance (Radwag Balances and Scales, Radom, Poland) and dissolved sequentially in deionized water.

The second solution was prepared using soil filtrate. The soil sample was taken from a depth of approximately 1.5 m in the area where the DN 300 carbon steel gas pipeline connects to fittings made of 1.4301 steel. The gas pipeline and its fittings are protected against corrosion by a cathodic protection system. The collected soil samples were then prepared by weighing 100 g of soil on a RADWAG^®^ WLC 6/C1/R scale and adding one liter of deionized water to them. The three solutions prepared in this way were left for a week to allow all the salts contained in the soil to dissolve completely.

Both solutions were tested to determine their pH, conductivity, and chloride concentration in the soil filtrate solution. A VWR^®^ pHenomenal^®^ MU 6100H meter (VWR, Radnor, PA, USA) was used to determine the conductivity and pH of the solutions. An Elmetron^®^ buffer solution with a pH of 7 was used to calibrate the meter. The measurements were performed at a temperature of 19.2 °C. The obtained results of solution conductivity in μS/cm were converted to Ωm. In order to determine the chloride concentration, part of the obtained solution was decanted into test tubes to be placed in a centrifuge and thoroughly separated from the sediment. Eight samples were rotated for 5 min at 4000 revolutions per minute. After rotation, three samples of 100 mL of solution were prepared, in which chlorides were measured using the Mohr method. The chlorides were determined in the presence of 1 mL of a 10% potassium chromate (VI) solution against a 0.05 M silver nitrate (V) solution. Table 3 summarizes the parameters of both solutions.

### 2.2. Test Samples

Electrochemical tests were performed on four stainless steels, namely, 1.4301, 1.4401, 1.4512, 1.4125, and, for comparison, carbon steel S235. The chemical composition of the steels in accordance with EN 10088 [31] is presented in Table 4.

Steel 1.4301 has an austenitic structure, steel 1.4401 also has an austenitic structure, steel 1.4512 is titanium-stabilized ferritic steel, and steel 1.4125 has a martensitic structure. The chromium content of the samples used in the study is presented in Table 5.

The samples used in the study were cylindrical in shape and equipped with an electrical connector. They were embedded in resin in such a way that only the base remained in contact with the electrolyte. The exposed area of the samples was 4.7 cm^2^. Before polarization began, each sample was cleaned by grinding with 500- and 1500-grit sandpaper, and then degreased with acetone. After cleaning, the samples were left to rest to restore the properties of the passive layer.

### 2.3. Electrochemical Tests

**Figure 1:**
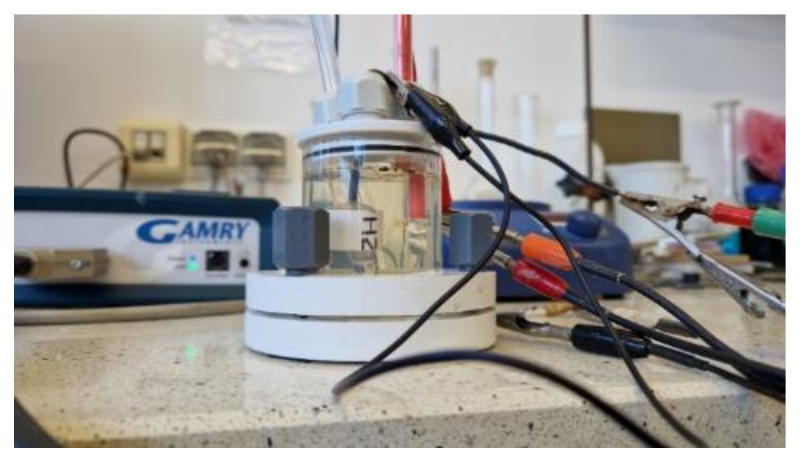
System for electrochemical measurements.

Potentiodynamic curves for large cathodic polarization and Tafel plots were determined for all tested materials in prepared soil solutions. The tests were conducted at a temperature of about 22 °C. Electrochemical measurements were carried out in a three-electrode system. The working electrode (WE) was stainless steel 1.4301, 1.4401, 1.4512, and 1.4125, and carbon steel S235. A saturated calomel electrode (SCE) was used as a reference electrode (RE), and platinum mesh as a counter electrode (CE). Measurements and data analysis were performed using a Gamry 1000^TM^ system (Gamry Instruments, Warminster, PA, USA). Figure 1 shows the measuring station during an exemplary electrochemical measurement. Table 6 summarizes the operating parameters of the device during the tests.

### 2.4. Exposure of Samples Under Cathodic Polarization Conditions

The samples of stainless steels 1.4301 and 1.4401 were cathodically polarized at a set potential (potentiostatic mode) in the prepared solutions for a period of one month. The corrosion rate was determined based on their mass loss. The plates of each steel grade, with dimensions of 75 mm by 45 mm, were cut from 0.8 mm thick metal sheet, and then a wire made of 1.4301 steel was welded to them to create an electrical connection.

The samples were polarized to a potential of −0.4 V vs. SCE, which corresponds to the protection potential of stainless steels specified in the EN 12954 standard. In the second experiment, the applied cathodic protection potential was −1.1 V vs. SCE. This value corresponds to the limit potential given in EN 12954 for the polarization of carbon steel. Comparatively, the samples that were not polarized were exposed in the same solutions. Before the exposure, all samples were weighed after being degreased with acetone. Weighing was performed using a RADWAG AS 220/C/2 analytical balance.

**Figure 2:**
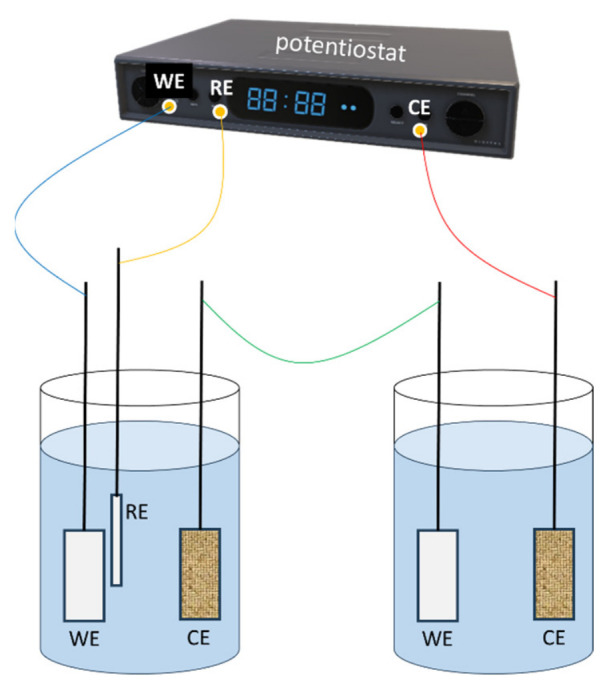
Diagram of the measuring set: WE—working electrode (steel sample); CE—counter electrode (titanium mesh); RE—reference electrode (SCE).

A diagram of the measuring set is shown in Figure 2. An Atlas Sollich 0931 potentiostat (manufacturer: ATLAS-SOLLICH ZSE Sp. z o. o., Rębiechowo, Poland) was used for polarization. Two identical measurement cells were connected in series with the potentiostat. This made it possible to polarize two steel samples at the same applied potential simultaneously. The reference electrode was an SCE, and the polarization anode (counter electrode) was a titanium mesh.

## 3. Results and Discussion

### 3.1. Electrochemical Fundamentals of Cathodic Polarization

The cathodic protection process involves imposing a protection potential value on the structure. This potential is determined by applying a specific cathodic overpotential to the structure [32]. The overpotential is the difference between the protection potential and the corrosion potential of the structure [33]. Once the polarized electrode exceeds a certain potential value, the overpotential applied to the electrode leads to a state in which other thermodynamic reactions can occur. In the case of cathodic polarization, one such reaction is the formation of hydrogen gas [34].

The corrosion process can be described using the Butler–Volmer equation (Equation (1)) [35].

(1) $$i=i_{corr}\left[exp\left(\frac{\eta }{\beta_{a}}\right)-exp\left(-\frac{\eta }{\beta_{k}}\right)\right]$$

where i is the global current density of electrode reaction, $i_{corr}$ is the corrosion current density, η is the overpotential at the electrode, and $\beta_{a},\;\beta_{k}$ are the anodic and cathodic Tafel coefficients [36]. An active cathodic protection system imposes high overpotential values directed towards the cathodic reaction. In the case of large negative overpotentials, when *η* > 0.1 V, we can simplify Equation (1) to Equation (2):

(2) $$i=i_{k}=-i_{corr}exp\left(-\frac{\eta }{\beta_{k}}\right)$$

where, in this case, the total reaction current at the electrode takes the value of the cathodic current density $i_{k}$ [37].

The formation of molecular hydrogen on the electrode surface depends on the following factors: electrode material, electrolyte environment, applied overpotential, and temperature. The first stage of the electrode reaction is the transport of water molecules to the electrode surface [38]. Depending on the environment, it takes different forms. In an acidic environment, the hydroxonium ion *H_3_O^+^* is adsorbed on the surface (see Equation (3)), while in an alkaline and neutral environment, the *H_2_O* molecule is the adsorbing species (see Equation (4)).

(3) $$H_{3}O^{+}+e^{-}\to H_{ad}+H_{2}O$$

(4) $$H_{2}O+e^{-}\to H_{ad}+{OH}^{-}$$

The resulting atomic hydrogen is adsorbed on the metal surface. Depending on the local surface conditions, atomic hydrogen can combine to form a gaseous hydrogen molecule or diffuse into the metal crystal lattice to form non-metallic inclusions. Atomic hydrogen diffusing into the metal structure poses a major threat due to the phenomenon of hydrogen embrittlement and susceptibility to stress corrosion cracking.

### 3.2. Analysis of Electrochemical Test Results

In real corrosion systems, the hydrogen evolution reaction does not occur at the equilibrium potential due to the kinetic resistance of the process. It only begins at a certain overpotential (which varies for different steel grades), at a more electro-negative potential of the cathodically polarized steel.

**Figure 3:**
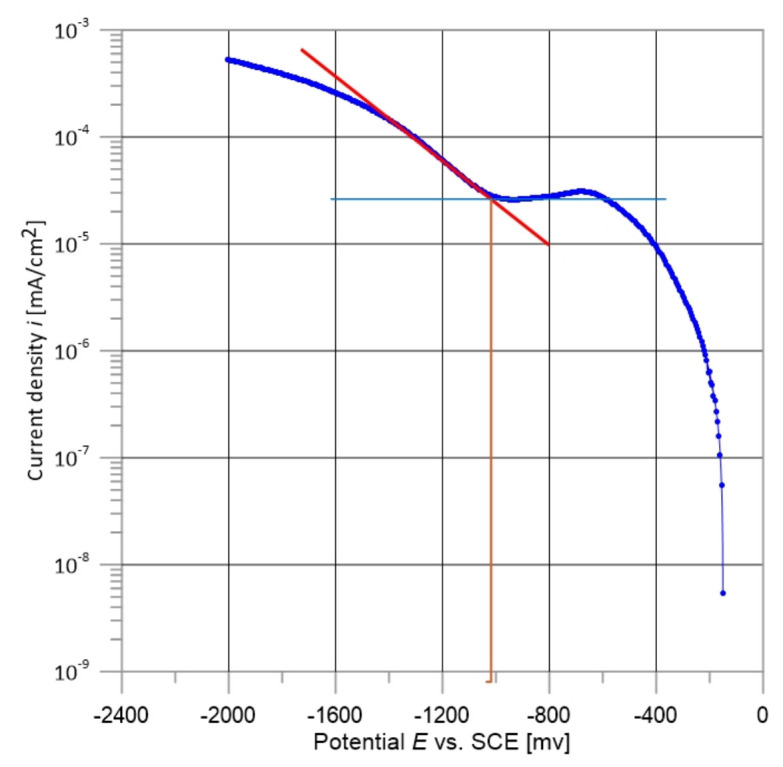
Illustration of the method for determining hydrogen evolution potential on the example of 1.4301 steel in artificial soil.

The hydrogen evolution potential on the steel surface was determined using a polarization curve for large cathodic polarization. Figure 3 illustrates how this potential was found using the example of 1.4301 stainless steel in artificial soil. The hydrogen evolution reaction occurs at potentials corresponding to the linear segment of the polarization curve (blue dotted line) plotted in the log(i) = f(E) coordinate system, as well as at lower potentials, and its rate depends on the cathodic polarization applied. The critical potential EH_2_ at which hydrogen evolution begins was determined at the point of intersection of two lines: the tangent to the linear segment (red line) and the tangent to the end of the segment associated with reduction reactions, including oxygen reduction (aquamarine line). The potential value for this point on the horizontal axis is indicated by the brown line.

**Figure 4:**
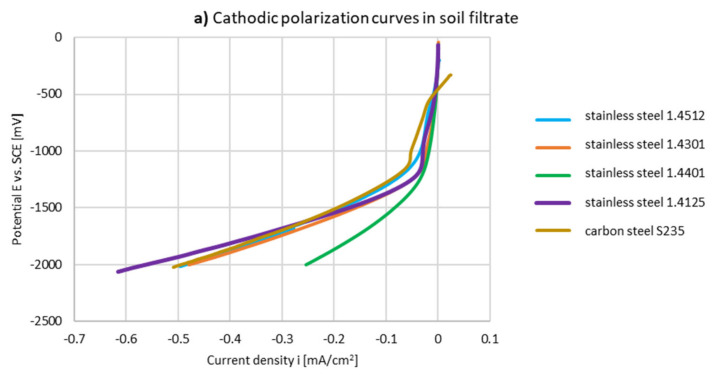

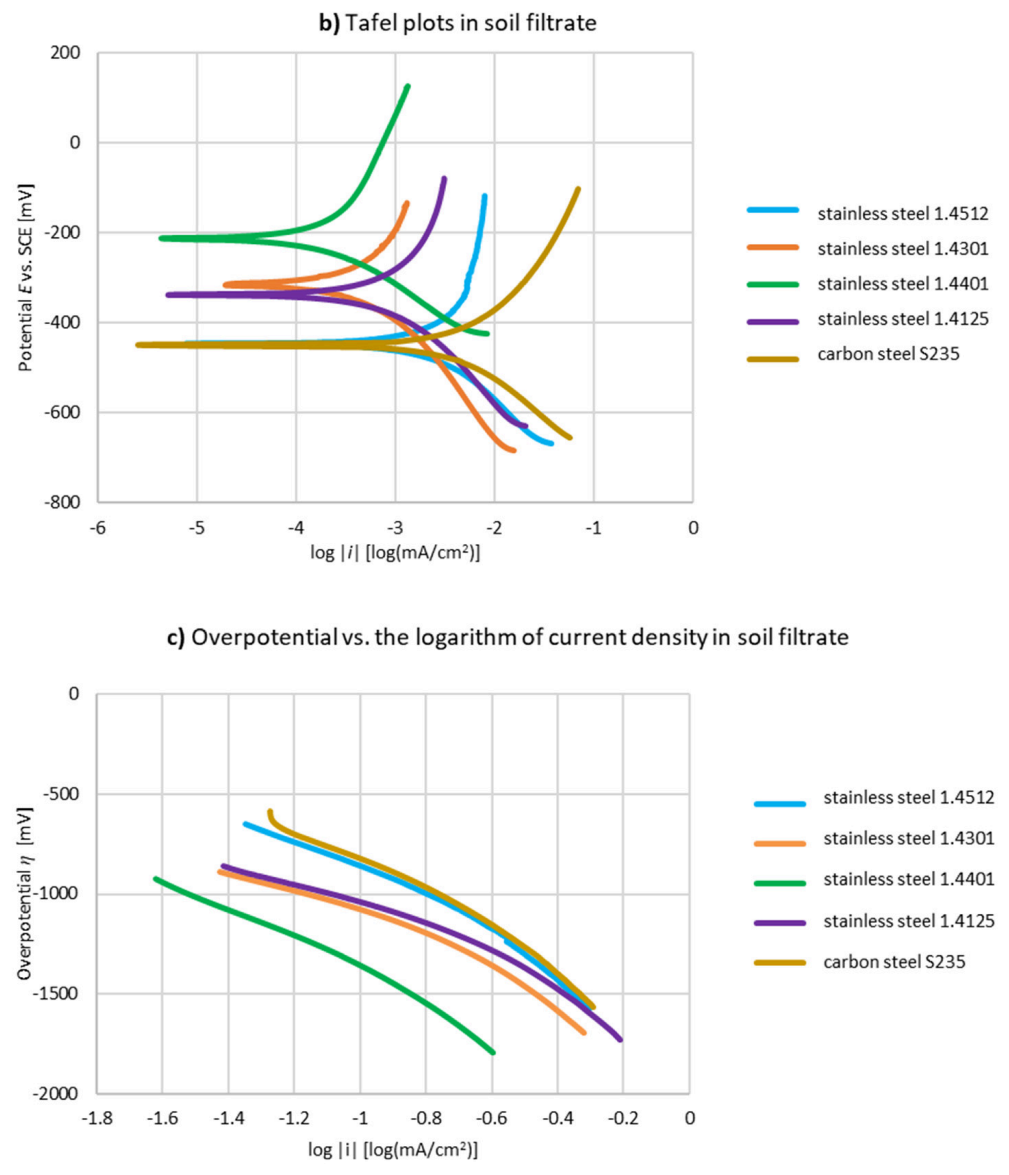
Electrochemical graphs of the tests in soil filtrate solution: (**a**) potentiodynamic curve, (**b**) Tafel plots, and (**c**) dependence of overpotential on the logarithm of current density.

**Figure 5:**
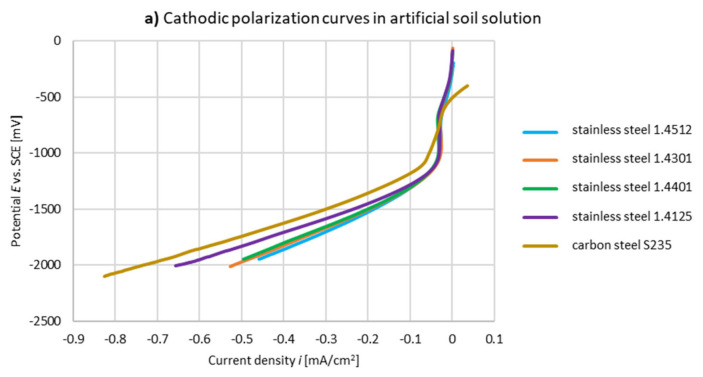

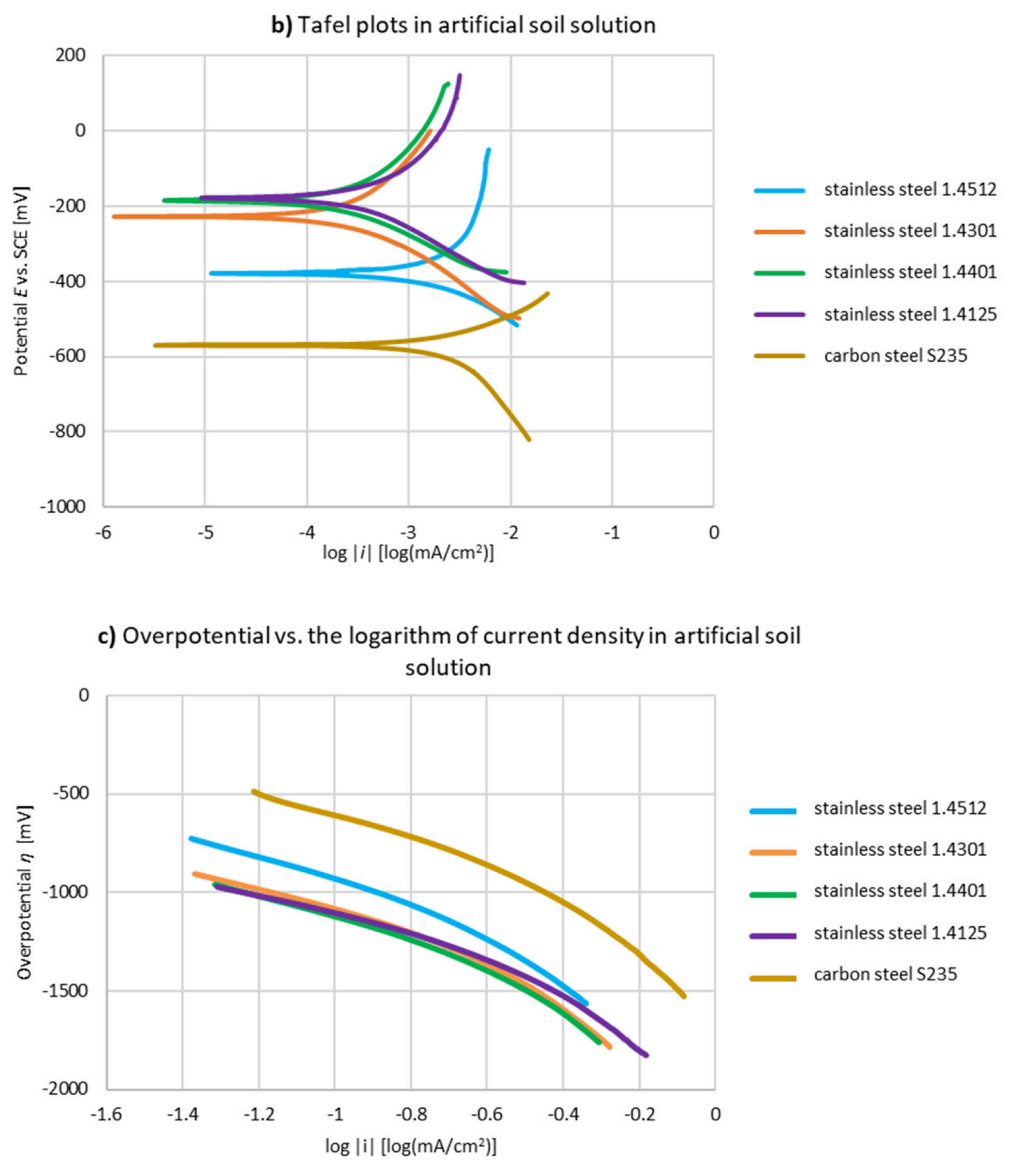
Electrochemical graphs of the tests in artificial soil solution: (**a**) potentiodynamic curve, (**b**) Tafel plots, and (**c**) dependence of overpotential on the logarithm of current density.

Figure 4 shows exemplary graphs of the results of electrochemical tests in a soil filtrate solution, and Figure 5 in an artificial soil solution. Cathodic polarization curves (Figure 4a and Figure 5a) were used to determine the critical potential. Based on the Tafel plots (Figure 4b and Figure 5b), the corrosion potential, corrosion current, and corrosion rate of the samples in a given environment were determined. Knowing the corrosion potential of steel, it is possible to determine the relationship between the overpotential and the logarithm of the current density (Figure 4c and Figure 5c). Based on the obtained curves, the critical value of the cathodic current density required to polarize the surface so that hydrogen gas is released was calculated. The fitted linear equation for each curve also allows for the calculation of the electrode reaction current density, which corresponds to the rate of hydrogen evolution at the steel surface.

The collected measurement results are summarized in Table 7. The determined potentials vs. SCE were converted to the copper sulfate electrode CSE to compare the obtained results with the values given in the EN 12954 standard, regarding the protection potential and critical potential of carbon steel: E_P_ = −850 mV and E_L_ = −1100 mV vs. CSE. The standard potential of a calomel electrode is 241 mV vs. normal hydrogen electrode (NHE), while that of a copper(II) sulfate electrode is 340 mV vs. NHE [39]. Therefore, the potential values relative to the CSE are 99 mV lower than those expressed relative to the SCE.

When comparing the corrosion potentials and corrosion rates of steel in both solutions, the higher corrosive aggressiveness of the soil filtrate compared to the artificial soil solution is noticeable. For all stainless steels, the difference in their corrosion potentials in both solutions is approximately 100 mV (more positive in artificial soil), with the exception of steel 1.4401, which has a similar potential in both environments. The lack of a significant difference in the corrosion potentials of this steel may be due to its more stable austenitic phase, resulting from the higher nickel content in the alloy.

The results obtained indicate that the hydrogen evolution potential value depends on the chromium content in the alloy. Furthermore, in more acidic soils (such as artificial soil—pH around 5.3), the hydrogen evolution potential on stainless steels is more electropositive (higher) than in neutral soils (such as soil filtrate—pH around 6.9). Steels for which this potential was the most negative in both solutions contain more than 16% of chromium. The hydrogen evolution potential for these steels was lower than that for carbon steel. For steel 1.4512 containing 12% of chromium, this potential in soil filtrate (−1075 mV) and in artificial soil (−1050 mV) was the most positive and was even higher than the hydrogen evolution potential for carbon steel (−1175 mV and −1180 mV, respectively).

The determined overpotential values, at which hydrogen evolution begins, range in artificial soil for the tested stainless steels from about −570 mV to −890 mV. Steel 1.4401 exhibits a similar overpotential value regardless of the environment (about −890 mV), which is due to its superior corrosion resistance. When the chromium content is about 12%, as in the 1.4512 steel, the overpotential value is only −570 mV. The significantly higher overpotential values for stainless steels containing more than 16% of chromium, compared with carbon steel, demonstrate how the presence of a passive layer affects the critical hydrogen evolution potential.

The critical values of the protection current density (current polarizing to the hydrogen evolution potential) in soil filtrate are lower than in artificial soil solution. In the first solution, the values range from 0.225 A/m^2^ to 0.32 A/m^2^ for all tested stainless steels, and 0.54 A/m^2^ for carbon steel. In the second solution, the values for stainless steels range from 0.30 A/m^2^ to 0.38 A/m^2^, and 0.65 A/m^2^ for carbon steel. The differences between individual steels result from the value of charge transfer through the dielectric layer on the electrode surface and the corrosive environment, which affects the value of the hydrogen evolution overpotential. This results in a significant difference in values between stainless steels and carbon steel, which has no passive layer limitation on its surface, allowing for a higher cathodic current density. Thus, lower cathodic current densities will polarize the stainless steel electrode to the hydrogen evolution potential.

### 3.3. Analysis of Exposure Results Under Cathodic Polarization Conditions

Table 8 shows the corrosion rate of polarized samples of 1.4301 and 1.4401 steel. The values of corrosion rate for both solutions are higher for polarized 1.4301 steel than for 1.4401 steel. This results from the higher corrosion resistance of the 1.4401 steel compared to 1.4301. For steel polarized to a value of −1.1 V, no corrosion was observed in either steel grade in either solution.

Table 9 shows the corrosion rate of non-polarized samples of 1.4301 and 1.4401 steel. The average corrosion rate of the 1.4301 steel in a soil filtrate solution is 0.0007 mm/year, and in an artificial soil solution 0.0009 mm/year. For the 1.4401 steel, the average corrosion rate in a soil filtrate solution is 0.0005 mm/year, and in an artificial solution 0.0014 mm/year. Low corrosion rates regardless of the type of steel and exposure environment indicate the high resistance of these materials in the tested environments, and no difference between the choice of steel as a structural material in the soil conditions.

It is worth noting that samples polarized to the potential of −0.4 V vs. SCE exhibit higher corrosion rates than non-polarized steels. The values are similar for both steel grades, and the corrosion rates are higher for polarized steel in the artificial soil solution than in the filtrate. Non-polarized steels have approximately three–four times lower corrosion rates than polarized steels. Despite applying the limit value of the protection potential for stainless steels according to the EN 12954 standard, the corrosion rates are higher for polarized than for non-polarized steels. This may be due to the violation of the passive layer of stainless steels. This leads to the conclusion that an incorrectly selected potential value during the cathodic polarization of stainless steels causes a greater corrosion risk than in the case of no polarization and no violation of the passive layer of stainless steels.

## 4. Application of the Obtained Results to an Actual Cathodic Protection System

The values of critical hydrogen evolution potentials obtained for stainless steels containing more than 16% of chromium (grades 1.4301, 1.4401, and 1.4125) are more negative than the critical hydrogen evolution potential for carbon steel. This suggests that, under actual soil conditions, the critical potential value E_L_ specified in EN 12954 for carbon steel (−1100 mV vs. CSE) does not pose a risk to stainless steels. At this level of cathodic polarization, there is no risk of hydrogen evolution on the surface of stainless steel.

In the case of steel containing 12% of chromium (such as 1.4512), the hydrogen evolution potential (about −1050 mV) is close to the E_L_ potential, so the level of cathodic polarization of underground structures made from this steel should be selected with care.

If, in a complex structure (carbon steel + stainless steel), it is not possible to directly measure the potential of the stainless steel section under polarization, the risk of hydrogen evolution on the surface of the stainless steel can be determined based on the cathodic protection current density. It can be determined by knowing the protection current drawn by that section and its surface area. Not exceeding the critical value of the current density, determined for a given environment in the same manner as in this study, will not cause hydrogen evolution on the surface of the stainless steel. For example, in the case of the soil tested in this study and a structure made of 1.4301 steel buried in it, the cathodic protection current density should not exceed 0.305 A/m^2^ (Table 7).

A separate issue concerns the protection potential of stainless steel in soil, specified by the EN 12954 standard as −0.45 V vs. CSE. In the tests conducted in this study, such polarization resulted in a higher corrosion rate than that of unprotected steel (reference sample). In the environments studied in this work—slightly acidic (artificial soil) and neutral (soil filtrate)—the protection potential of stainless steel should be significantly more negative. On the other hand, the corrosion rates of the polarized steels were significantly lower than 0.01 mm/year, which is the threshold adopted in the EN 12954 standard for the effect of fully effective cathodic protection. However, the results obtained call into question the rationale for intentionally polarizing stainless steels in low-aggressive environments.

## 5. Conclusions

- The values of critical hydrogen evolution potentials are more negative with increasing chromium content in stainless steels.
- The critical hydrogen evolution potentials for stainless steels containing more than 16% of chromium (grades 1.4301, 1.4401, and 1.4125) are more negative than the critical potential for carbon steel (about −1.18 V vs. CSE). All these values are lower than the critical potential of permissible cathodic polarization of carbon steel specified in the standard EN 12954 (−1.1 V vs. CSE).
- Cathodic polarization of a complex structure (carbon steel + stainless steel) to a potential within the range recommended for carbon steel, i.e., from −0.85 V to −1.1 V vs. CSE, does not pose a risk to stainless steel containing a high chromium content, since hydrogen does not evolve on the stainless steel surface at these potentials.
- The critical hydrogen evolution potential for stainless steel containing 12% of chromium (grade 1.4512) is equal to −1050 mV in slightly acidic soil (pH 5.3), and −1075 mV in neutral soil. These values are close to the permissible cathodic polarization of carbon steel specified in the standard EN 12954, so the level of cathodic polarization of underground structures made from this steel should be selected with care.
- The critical protection current density for stainless steel in an artificial soil environment is approximately 0.30–0.38 A/m^2^, depending on the grade of steel, and is lower than that for carbon steel, which is approximately 0.65 A/m^2^.
- In soils with low corrosion aggressiveness, cathodic protection with incorrectly selected polarization parameters may destroy the passive layer on the surface of stainless steel.

The presented research results confirm that the application of cathodic protection to stainless steel structures in natural environments is justified but requires appropriate and precise selection of the operating parameters of the protection system depending on environmental conditions. Potential directions for future research may involve determining the influence of electrolyte salinity, resistivity, oxygenation, and pH on the protection potential of stainless steels. Such research would enable the determination of a universal range of potential values, from the threshold of full corrosion protection to that of hydrogen evolution. Another important issue requiring further research is the effect of soil leaching—especially of acidic soil—on the stability of the passive layer on stainless steel, as well as the values of the protection potential and hydrogen evolution potential under such conditions.

## Figures and Tables

**Table 1:** Selection of stainless steel depending on the environment.

Location	Environment Parameter	Parameter Value	Stainless Steels
Inland	Cl^−^	<500 ppm	1.4301; 1.4307;
	Resistivity	>10 Ωm	1.4401; 1.4404;
	pH	>4.5	1.4521
Chlorides—non-tidal zone	Cl^−^	<1500 ppm	
	Resistivity	>10 Ωm	1.4401; 1.4404
	pH	>4.5	
Chlorides—tidal zone	Cl^−^	<500 ppm	
	Resistivity	>10 Ωm	1.4410; 1.4547; 1.4529
	pH	>4.5	

**Table 2:** Composition of the 5 dm^3^ artificial soil solution.

Component	Amount [mg]
CaCl_2_∙2H_2_O	36.76
KNO_3_	15.15
Na_2_SO_4_	8.522
NH_4_NO_3_	26.41
MgSO_4_∙7H_2_O	24.65

**Table 3:** Concentration, resistivity, and pH values of the solutions used in the experiments.

Parameter	Artificial Soil Solution	Soil Filtrate Solution
Concentration Cl^−^ [ppm]	36.76	3.55
Resistivity [Ωm]	64.35	177.62
pH	5.295	6.932

**Table 4:** Normalized chemical composition (in wt%) of steel grades.

Grade	C	Si	Mn	P	S	Cr	Ni	N	Fe
1.4301	≤0.07	≤1.00	≤2.00	≤0.045	≤0.015	17.5–19.5	8.0–10.5	≤0.11	bal.
1.4401	≤0.07	≤1.00	≤2.00	≤0.045	≤0.015	16.5–18.5	10.0–13.0	≤0.11	bal.
1.4512	≤0.03	≤1.00	≤1.00	≤0.040	≤0.015	10.5–12.5	-	≤0.03	bal.
1.4125	0.95–1.20	≤1.00	≤1.00	≤0.040	≤0.030	16.0–18.0	-	-	bal.
S235	≤0.17	≤1.40	≤1.40	≤0.035	≤0.035	-	-	≤0.012	bal.

**Table 5:** Chromium content (in wt%) in the tested samples.

Steel Grade	Cr
1.4301	18%
1.4401	16.5%
1.4512	12%
1.4125	17%

**Table 6:** Operating parameters of the measuring device.

Parameter	Potentiodynamic Curves	Tafel Plots
Initial potential [V]	0.1 vs. SCE	0.25 vs. corrosion potential
Final potential [V]	−2.0 vs. SCE	−0.25 vs. corrosion potential
Scan rate [mV/s]	1	1
Sampling period [s]	1	1
Sample surface [cm^2^]	4.7	4.7
Steel density [g/cm^3^]	7.8	7.8

**Table 7:** Average results of electrochemical measurements. Potentials given vs. CSE.

Solution	Steel	Corrosion Potential *E_corr_* [mV]	Potential for Hydrogen Evaluation *E_H2_* [mV] ±5 mV	Overpotential *η* [mV] ±5 mV	Corrosion Current Density *i_corr_* [A/m^2^]	Critical Protection Current Density *i_cr_* [A/m^2^]	Calculated Corrosion Rate *v_corr_* [mm/year]
Soil filtrate	1.4301	−409	−1250	−841	0.032	0.305	0.0007
	1.4401	−313	−1195	−882	0.020	0.225	0.0002
	1.4125	−435	−1225	−790	0.033	0.320	0.0009
	1.4512	−545	−1075	−530	0.100	0.260	0.0025
	S235	−551	−1175	−624	0.123	0.540	0.0051
Artificial soil	1.4301	−323	−1105	−782	0.043	0.375	0.0004
	1.4401	−283	−1175	−892	0.021	0.365	0.0002
	1.4125	−278	−1140	−862	0.036	0.380	0.0004
	1.4512	−478	−1050	−572	0.064	0.300	0.0019
	S235	−671	−1180	−509	0.235	0.650	0.0036

**Table 8:** Average corrosion rate of polarized steel samples.

Solution	Stainless 1.4301 Corrosion Rate		Stainless 1.4401 Corrosion Rate	
	at Potential −0.4 V	at Potential −1.1 V	at Potential −0.4 V	at Potential −1.1 V
Soil filtrate	0.0022 mm/y	0	0.0019 mm/y	0
Artificial soil	0.0033 mm/y	0	0.0024 mm/y	0

**Table 9:** Average corrosion rate of non-polarized steel samples.

Solution	Stainless 1.4301 Corrosion Rate	Stainless 1.4401 Corrosion Rate
Soil filtrate	0.0007 mm/y	0.0005 mm/y
Artificial soil	0.0009 mm/y	0.0014 mm/y

## Data Availability

The original contributions presented in the study are included in this article; further inquiries can be directed to the corresponding author.

## References

[1] Bai J., Li H., Wang G. (2025). Review on Evolution of Oxide Layer and Chromium Depletion Layer of Austenitic Stainless Steel during Industrial Processing. Steel Res. Int..

[2] Walport F., Kucukler M., Gardner L. (2022). Stability Design of Stainless Steel Structures. J. Struct. Eng..

[3] (2015). Designation Systems for Steels—Part 2: Numerical System.

[4] Żakowski K., Szociński M., Krakowiak S. (2025). Assessment Methods for DC Stray Current Corrosion Hazards in Underground Gas Pipelines: A Review Focused on Rail Traction Systems. Energies.

[5] Wang C., Xu S., Wang Y., Song A., Ding W., Li W., Qin G. (2026). Towards Understanding Hydrogen Embrittlement under Stray Current Interference through a Quantitative Method Based on Multifractal Characteristics. J. Mater. Sci. Technol..

[6] Liu M., Ni Z., Du C., Liu Z., Sun M., Fan E., Wang Q., Yang X., Li X. (2021). Failure Investigation of a 304 Stainless Steel Geothermal Tube. Eng. Fail. Anal..

[7] Feng X., Yan Q., Zhu C., Chen Z., Lu X., Lu S. (2022). Corrosion Performance of 201 Low-Nickel Stainless Steel Anchor in Cl^–^ Contaminated Underground Water with Various Concentrations of SO_4_^2−^ and HCO_3_^−^. J. Electrochem. Soc..

[8] Tawancy H.M., Al-Hadhrami L.M. (2012). Case Study: Pitting and Stress Corrosion Cracking in Heat-Affected Zone of Welded Underground 304 Stainless Steel Pipe. J. Mater. Eng. Perform..

[9] Farh H.M.H., Ben Seghier M.E.A., Zayed T. (2023). A Comprehensive Review of Corrosion Protection and Control Techniques for Metallic Pipelines. Eng. Fail. Anal..

[10] Cunat P.-J., Charles J. (2004). Stainless Steel, from a Century to the Next. Rev. Met..

[11] Angst U.M. (2019). A Critical Review of the Science and Engineering of Cathodic Protection of Steel in Soil and Concrete. Corrosion.

[12] Mahlobo M.G.R., Olubambi P.A., Jeannin M., Refait P. (2020). Cathodic Protection of Buried Steel Structures: Processes Occurring at the Steel/Soil Interface during Wet/Dry Cycles. Mater. Corros..

[13] Wang C., Wang Y., Xu S., Song A., Ding W., Li W., Wang S. (2025). Effect of Stray Current Interference on Hydrogen Embrittlement Sensitivity of Q235 Pipeline Steel Considering Local Deformation on the Steel Surface and Environmental pH Value. Constr. Build. Mater..

[14] Wang Y., Wang C., Xu S., Li W., Ding W., Li M. (2025). Study on Hydrogen Permeation Behavior of Q235 Pipeline Steel under Stray Current Interference from Urban Rail Transit System. Anti-Corros. Methods Mater..

[15] Abebe B.A., Altuncu E. (2024). A Review on Hydrogen Embrittlement Behavior of Steel Structures and Measurement Methods. Int. Adv. Res. Eng. J..

[16] Ghosh G., Rostron P., Garg R., Panday A. (2018). Hydrogen Induced Cracking of Pipeline and Pressure Vessel Steels: A Review. Eng. Fract. Mech..

[17] Findley K.O., Lawrence S.K., O’Brien M.K. (2022). Engineering Challenges Associated With Hydrogen Embrittlement in Steels. Encyclopedia of Materials: Metals and Alloys.

[18] Wu C., Yan C., Zhang S., Zhou L., Shen M., Tian Z. (2024). Research on Hydrogen-Induced Induced Cracking Sensitivity of X80 Pipeline Steel under Different Heat Treatments. Materials.

[19] Javeria U., Kim S.J. (2025). Investigation of Hydrogen Embrittlement in Steel Alloys: Mechanism, Factors, Advanced Methods and Materials, Applications, Challenges, and Future Directions: A Review. J. Mater. Res. Technol..

[20] Zhang P., Laleh M., Hughes A.E., Marceau R.K.W., Hilditch T., Tan M.Y. (2023). A Systematic Study on the Influence of Electrochemical Charging Conditions on the Hydrogen Embrittlement Behaviour of a Pipeline Steel. Int. J. Hydrogen Energy.

[21] Weber G., Merten B.J., Torrey J.D. (2019). Comparison of Cathodic Disbondment Test Methods. Mater. Perform..

[22] Narozny M., Zakowski K., Darowicki K. (2018). Application of Electrochemical Impedance Spectroscopy to Evaluate Cathodically Protected Coated Steel in Seawater. Constr. Build. Mater..

[23] (2019). Cathodic Protection of Buried or Immersed Metallic Structures—General Principles and Application for Pipelines.

[24] (2012). Cathodic Protection of Steel in Concrete.

[25] (2024). Control of External Corrosion on Underground or Submerged Metallic Piping Systems.

[26] Googan C. (2021). The Cathodic Protection Potential Criteria: Evaluation of the Evidence. Mater. Corros..

[27] (2005). Cathodic Protection of Complex Structures.

[28] Narozny M., Zakowski K., Darowicki K. (2017). Time Evolution of Electrochemical Impedance Spectra of Cathodically Protected Steel in Artificial Seawater. Constr. Build. Mater..

[29] Omura T., Nakamura J. (2011). Hydrogen Embrittlement of Stainless Steel. Corros. Eng..

[30] Ritchie J.M., Cresser M., Cotter-Howells J. (2001). Toxicological Response of a Bioluminescent Microbial Assay to Zn, Pb and Cu in an Artificial Soil Solution: Relationship with Total Metal Concentrations and Free Ion Activities. Environ. Pollut..

[31] (2024). Stainless Steels—Part 1: List of Stainless Steels.

[32] Revie R.W., Uhlig H.H. (2008). Corrosion and Corrosion Control: An Introduction to Corrosion Science and Engineering.

[33] Plieth W. (2008). Electrochemistry for Materials Science.

[34] Gong K., Sun D., Liu X., Li J., Wu M., Hu M. (2025). Effects of Hydrogen and Strain Rate on Stress Corrosion Cracking Mechanism of High Strength Pipeline Steel. Mater. Today Commun..

[35] Marcus P. (2011). Corrosion Mechanisms in Theory and Practice.

[36] Talbot D.E.J., Talbot J.D.R. (2018). Corrosion Science and Technology.

[37] Schütze M. (2002). Corrosion Books: Handbook of Corrosion Engineering. By Pierre R. Roberge—Materials and Corrosion 4/2002. Mater. Corros..

[38] Cramer S.D., Covino B.S. (2003). Corrosion: Fundamentals, Testing, and Protection.

[39] Haynes W.M. (2011). CRC Handbook of Chemistry and Physics.

